# Supplementary material for: Angelica sinensis polysaccharide as potential protectants against recurrent spontaneous abortion: focus on autophagy regulation
Source: Front Med (Lausanne). 2025 Jan 15;12:1522503. doi: 10.3389/fmed.2025.1522503 (PMC11774876; doi:10.3389/fmed.2025.1522503)
Supplement: Supplementary material S1 — The main instruments used during the LC-MS process, along with their models/specifications and manufacturers. [file Supplementary_file_1.zip › Metabolomics sequencing data FC1.2/4.多元统计分析/其他图形/Splot-PLS-DA(Exposure_Ctrl).pdf]

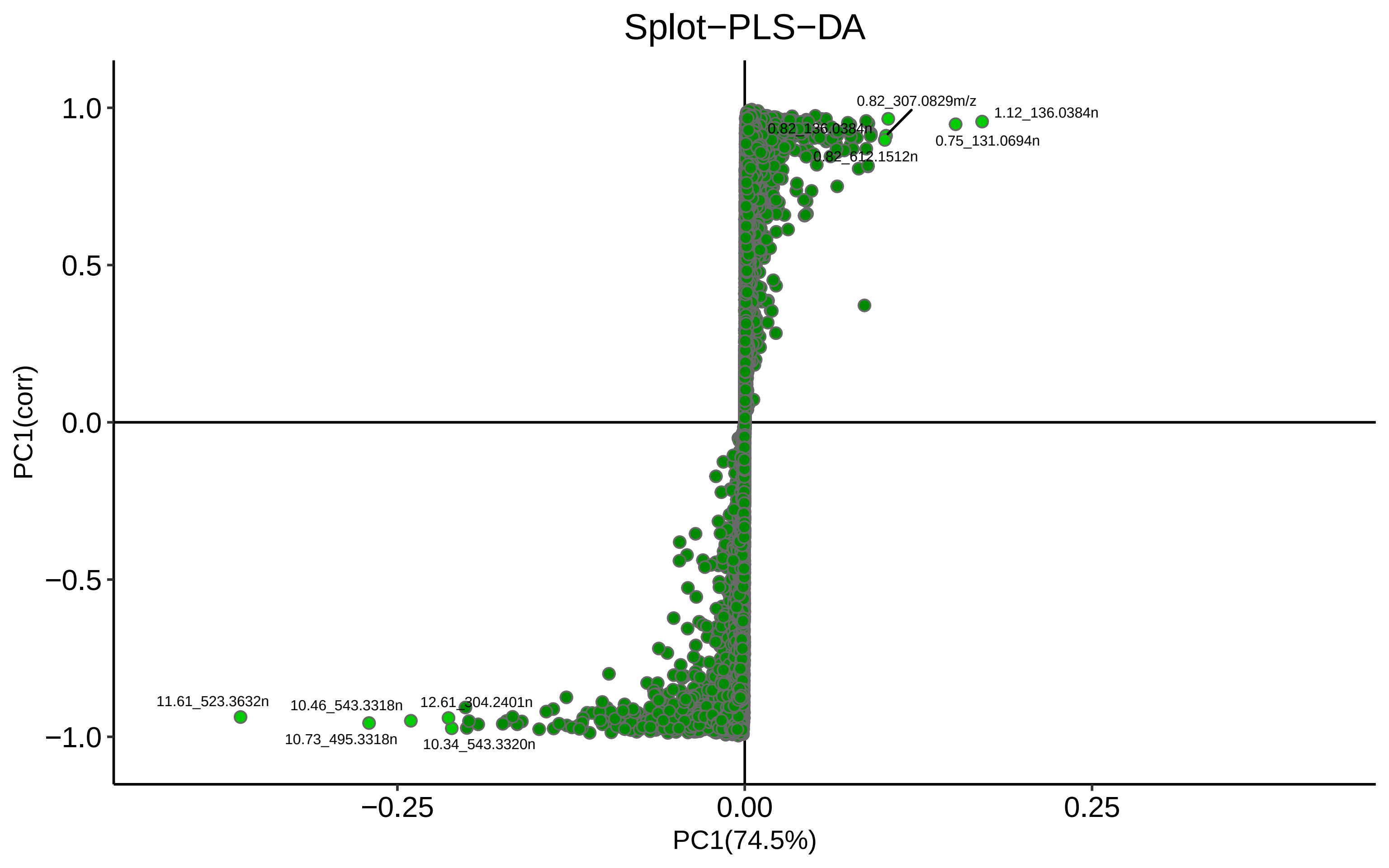

Splot−PLS−DA
PC1(corr)
PC1(74.5%)
1.0
0.5
0.0
−0.5
−1.0
−0.25
0.00
0.25
0.82_307.0829m/z
1.12_136.0384n
0.82_136.0384n
0.75_131.0694n
0.82_612.1512n
11.61_523.3632n
10.46_543.3318n
12.61_304.2401n
10.73_495.3318n
10.34_543.3320n
